# Does Simulation Training Improve the Accuracy of Vaginal Assessment of Labour Progress?

**DOI:** 10.7759/cureus.16089

**Published:** 2021-07-01

**Authors:** Girija Swaminathan, Shashank V Parulekar

**Affiliations:** 1 Obstetrics and Gynaecology, King Edward Memorial Hospital and Seth Gordhandas Sunderdas Medical College, Mumbai, IND

**Keywords:** simulation training, vaginal examination, labour progress assessment, skills training, cervical assessment

## Abstract

Aim

To measure the utility of the Simulation training model for training purposes over and above conventional methods of training for vaginal assessment during labour.

Methods

The study group included undergraduate trainees, and the control group included postgraduate trainees and qualified personnel, i.e. senior registrars and consultants. Participants from the study group were trained for vaginal assessment on the simulation training model. Then both the groups were tested on the model for accuracy in estimating each value of cervical dilatation and fetal station. Mean cervical dilatation and station accuracy scores were noted, and comparative analysis was done between the study and control groups.

Results

A total of 150 participants were included. The overall mean dilatation and station accuracy scores of a model trained study group participants were better than subjectively trained control group participants. Study group participants showed greater accuracy for smaller dilatations, i.e. 1, 2, 3, 4cm and middle dilatation, i.e. 5cm and 6cm (p value=<0.05). In contrast, comparing the two groups for higher dilatations from 6 to 10 cm did not show any statistical significance. Study group participants also showed greater accuracy for all the fetal stations except stations 0 and +1.

Conclusions

The simulation training model can be considered an in vitro training device to improve the trainees' understanding of cervical dilatation and fetal station and can be made a part of a routine obstetric teaching program.

## Introduction

Progress of labour can be monitored clinically by abdominal palpation and vaginal examination, out of which vaginal examination is the most accepted method of measuring labour progress. Dilatation of cervical os measured by digital vaginal examination can be used as a sole measure of labour progress. However, it is usually combined with other clinical observations like position and consistency of cervix and the position and level of descent of the fetal head in the maternal pelvis to aid decision making on labour progress [1]. The two main objective criteria used in vaginal examination to assess labour progress are cervical dilatation and descent of the fetal presenting part. Assessment of cervical dilatation in centimetres is one of the most critical aspects of vaginal examination during labour. Very few studies have evaluated the ability of obstetricians to judge cervical dilatation [2,3]. There is much inter-observer variation. The width of fingers assesses conventionally cervical dilatation. The size of the fingers vary, and so also the number of fingers expressing cervical dilatation. Knowledge of cervical dilatation in centimetres is an essential prerequisite of plotting of cervicograph, a graphical representation of cervical dilatation and descent of fetal head on the partogram.

Partograph is the most widely used tool by healthcare professionals for labour monitoring and is recommended by the World Health Organization (WHO) for use in the active stage of labour [4]. Cervicographic monitoring of labour can detect delayed progress timely for augmentation and referral of delivering woman for appropriate intervention. A graphic record of labour increases the quality of regularity of observations on the mother and fetus, provides early warning for the abnormal progress and assists in an early decision for referral, interventions and termination of labour [5]. Usually, postgraduate students learn vaginal examination and cervical assessment directly on patients. This kind of conventional training is not methodical. Also, repeated examinations done by students for learning purposes cause inconvenience and discomfort to patients and increase the rates of infection. There are no absolute norms that are accurate, and students tend to believe that the findings by seniors are correct, which are not necessarily true. Hence, there is a need for gold standard actual measurements which can be verified by any neutral observer and a model to assess the level of accuracy among various trainees and qualified personnel. Simulation training models are currently used in various specialities for undergraduate and postgraduate training, and they are one of the best teaching methods. Though vaginal assessment training simulators are being used for training purposes in some countries, they are not routinely used in most developing countries. Our Simulation training model is one such model that is simple, economical yet very practical and can be used even in low resource settings. The objective of this study was to determine whether conventional training methods are adequate for training students by testing them on our simulation training model and to measure the utility of this device for training purposes over and above conventional methods.

## Materials and methods

This cross-sectional observational study was undertaken at a prominent medical teaching institute and tertiary care referral centre in India. The study was commenced after the approval of the Institutional Ethics Committee. As the study was a pilot study, the number of participants was calculated based on an available number of participants. We included 150 participants in our study, 75 each in the control and the study group. Written, informed, and valid consent was obtained from all participants.

The study group included undergraduate students without any prior clinical obstetric experience. They were taught on the simulation training model about assessing cervical dilatation and fetal station and were allowed to practice on the model adequately. The Control group included postgraduate trainees and qualified Obstetricians and Gynaecologists (senior registrars and consultants), who had learnt vaginal examination by clinical exposure and practice on patients.

All study records were kept confidential at all times, and each participant was assessed privately in the presence of only the study investigators. The participants were kept anonymous, and their results were not revealed to any other person. Recruitment of participants was based on inclusion criteria. Each participant from the study group had a minimum of two visits, i.e. 1^st^ for training and 2^nd^ for testing, and each participant from the control group had a single visit for testing. The training was provided by the principal investigator and co-investigator, who had adequate training about how to use the simulation training model. There were two modules. Module 1 was the "Training module", the duration of which was 1 hour for each participant. During this module, the Student was first taught about the basics of vaginal examination, the stages of normal labour and the method for assessing cervical dilatation and fetal station. This module also oriented the students about the simulation training model, and the trainer demonstrated how to assess cervical dilatation and fetal station. The students were then taught the same on the model and allowed to practice as many times as they desired for about one week. Module 2 was the "Testing module" where participants from both groups were tested on the model for their accuracy of the vaginal assessment.

**Figure 1:**
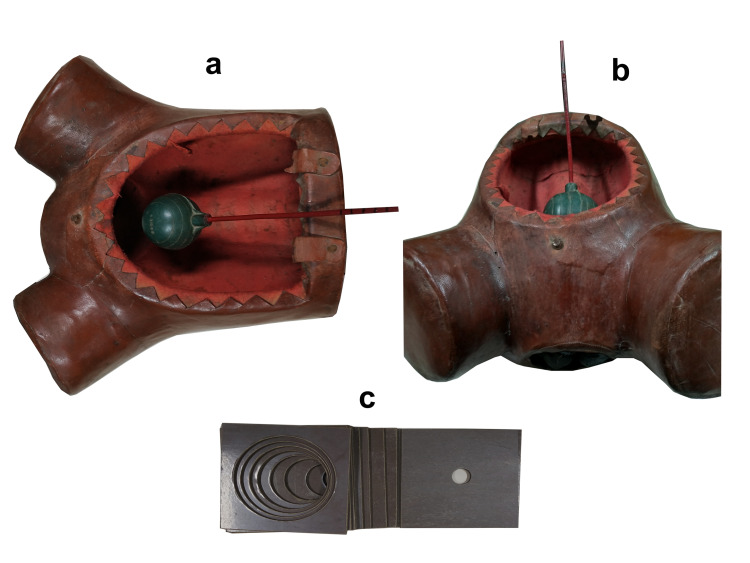
Simulation training model (a,b) Training model with fetal head rod, (c) Plates used for cervical dilatation assessment

The model for cervical dilatation consisted of a commercially designed mannequin which had place for vaginal examination and an abdominal opening through which locally designed and fabricated bakelite plates with circular central openings measuring 1 to 10cm in diameter could be placed perpendicular to the axis of the examining fingers. These were used for the assessment of cervical dilatation. Another part of the model included a locally designed and fabricated rod with a 10 cm diameter sphere representing the fetal head at its one end. There were two palpable markers on the inside of the mannequin at the level of ischial spines, which could be palpated during a simulated vaginal examination. The sphere was pushed downwards through the abdominal aperture of the mannequin, and the participant was asked to feel the leading point of the sphere by vaginal route. The participant could evaluate the station by estimating the level of the leading point of the sphere for the ischial spine level markers (station 0), being -5 to +5, each 1 cm above the ischial spine level being -1, and each 1 cm below the ischial spine level being +1. The long rod had 1 cm markings from -5 to +5 (Figure 1).

The plates used for assessment were placed randomly, varying sequence from 1 cm to 10 cm at the will of the assessor. Each plate was used only once. Similarly, the sphere was moved from -5 to +5 randomly at the will of the assessor. Participants were tested on the simulation training model to determine cervical dilatation and station of the fetal presenting part. The assessment method in our study was objective, and the assessment results were entered into the case record forms. Comparisons of accuracy between the study and control groups in vaginal assessments were carried out using the Student's t-test. Using the simulation training model's estimate as to the standard at each dilatation from 1 to 10 cm and station from -5 to +5, the mean estimate, with standard deviation, was calculated. Accuracy was defined as the participant's cervical dilatation and station estimate agreement with that of the model. For each measure of dilatation and station, the percentage of accurate participant assessments was calculated. Station assessment in centimetres from -5 through 0 up to +5 was tested. The p values of less than 0.05 were considered to indicate statistical significance.

## Results

There were 150 participants, with 75 each in the study group and control group, respectively. The distribution of the various participants based on their designation and years of experience in the assessment of cervical dilatation and the fetal station is shown in Table 1.

**Table 1:** Distribution of the various participants based on their designation and years of experience in vaginal assessment PG- Postgraduate, OG- Obstetrics & Gynaecology

DESIGNATION	EXPERIENCE ( IN YEARS )	NUMBER OF PARTICIPANTS
Undergraduate	0	75 (50%)
PG First year OG trainee	<1	11 (7.3%)
PG Second-year OG trainee	1-2	32 (21.3%)
PG Third year OG trainee	2-3	12 (8%)
Senior Registrar	3-5	9 (6%)
Assistant Professor	5-10	7 (4.67%)
Associate Professor	>10	4 (2.67%)

The participants were tested for each cervical dilatation from 1 cm to 10 cm in random order. Each participant was given a dilatation score based on the number of accurate observations made out of 10. Similarly, each participant was tested for every station value from -5 to +5, including station 0 and the station score was calculated out of 11 based on accurate observations. The average of the scores of various participants in each subgroup was calculated to get the mean dilatation and station scores (Table 2).

**Table 2:** Mean accuracy of participants in assessment of cervical dilatation and fetal station OG- Obstetrics & Gynaecology

DESIGNATION (Years of experience)	MEAN DILATATION SCORE	MEAN STATION SCORE
Associate Professor/Professor (>10 years)	7.33	8
Assistant Professor (5-10 years)	6.38	6.63
Senior Registrar (3-5 years)	5.1	3
Third-year OG trainee (2-3 years)	5.83	5.5
Second-year OG trainee (1-2 years)	5.16	4.81
First year OG trainee (<1 year)	5.73	5.1
Undergraduate student (0 years)	6.89	7.04

Our study results indicate that the performance of consultants, i.e. Professors/Associate Professors (mean dilatation score= 7.33 and mean station score=8) and Assistant Professors (mean dilatation score= 6.38 and mean station score= 6.63) was better than the Senior registrars and trainees (mean dilatation score= 5.36 and mean station score= 4.73) in terms of both dilatation and station assessment accuracy. It is also seen that the undergraduate students (mean dilatation score= 6.89 and mean station score= 7.04) who were trained on the simulation model had better accuracy in both cervical dilatation and station assessment compared to Senior registrars and trainees who had learnt by practising on patients. The study and control groups' dilatation and station accuracy scores were compared using an unpaired 't test. The trained model group showed higher mean scores than a subjectively trained group. The p-value in both dilatation and station was less than 0.0001, which showed that the difference was statistically significant (Table 3).

**Table 3:** Comparison of dilatation and station accuracy scores of study and control groups

GROUP	MEAN DILATATION SCORE	DILATATION STANDARD DEVIATION	DILATATION 95% CONFIDENCE INTERVAL	MEAN STATION SCORE	STATION STANDARD DEVIATION	STATION 95% CONFIDENCE INTERVAL
STUDY GROUP (MODEL TRAINED)	6.89	1.65	0.74-1.93	7.04	2.44	1.22-2.72
CONTROL GROUP (SUBJECTIVELY TRAINED)	5.56	2.02		5.07	2.21	
p value of Dilatation accuracy = <0.0001 and p value of Station accuracy = <0.0001						

The two groups were compared for each dilatation from 1 to 10 cm using the unpaired t-test to determine the group with higher accuracy in assessing cervical dilatation. The study group participants who were model trained showed greater accuracy for smaller dilatations, i.e. 1, 2, 3, 4cm and middle dilatations like 5cm and 6cm (p value= <0.05). In contrast, comparing the two groups for higher dilatations from 6 to 10 cm did not show any statistical significance (Table 4).

**Table 4:** Comparison of the two groups for various cervical dilatation values

DILATATION VALUE (IN CM)	STUDY GROUP MEAN	CONTROL GROUP MEAN	STUDY GROUP SD	CONTROL GROUP SD	95% CONFIDENCE INTERVAL	P VALUE
1	2.01	1.89	0.12	0.39	0.03 to 0.21	0.0113
2	2.01	1.89	0.12	0.39	0.03 to 0.21	0.0113
3	3	2.72	0.28	0.48	0.15 to 0.41	<0.0001
4	3.84	3.60	0.57	0.64	0.04 to 0.44	0.0162
5	4.76	4.37	0.61	0.71	0.17 to 0.60	0.0005
6	5.83	5.39	0.78	0.90	0.17 to 0.71	0.0016
7	6.80	6.51	0.99	0.92	-0.01 to 0.60	0.0617
8	7.83	7.84	0.79	0.97	-0.30 to 0.27	0.9269
9	8.95	8.65	0.85	1.08	-0.02 to 0.61	0.0675
10	9.64	9.52	0.63	0.72	-0.10 to 0.34	0.2801

The two groups were also compared for each fetal station value from -5 to +5. The study group participants showed greater accuracy than the subjectively trained group for all the fetal stations except stations 0 and +1, where there was no statistical significance between the results of the two groups (Table 5).

**Table 5:** Comparison of the two groups for various fetal station values SD- Standard deviation

FETAL STATION VALUE	STUDY GROUP MEAN	CONTROL GROUP MEAN	STUDY GROUP SD	CONTROL GROUP SD	95% CONFIDENCE INTERVAL	P VALUE
-5	-4.67	-4.17	0.58	0.83	-0.72 to -0.26	<0.0001
-4	-3.9	-3.41	0.73	0.93	-0.78 to -0.24	0.0003
-3	-3.03	-2.44	0.73	0.78	-0.83 to -0.34	<0.0001
-2	-1.95	-1.61	0.75	0.77	-0.58 to -0.09	0.0081
-1	-1.01	-0.76	0.80	0.81	-0.50 to -0.01	0.0420
0	0.01	0.15	0.65	1.01	-0.41 to 0.14	0.3371
+1	1.13	1.04	0.76	0.74	-0.15 to 0.34	0.4480
+2	2.00	1.72	0.72	0.78	0.04 to 0.52	0.0236
+3	2.83	2.56	0.81	0.78	0.01 to 0.52	0.0414
+4	3.81	3.43	0.56	0.86	0.15 to 0.62	0.0014
+5	4.77	4.12	0.51	0.84	0.43 to 0.88	<0.0001

We also assessed the percentage accuracy of the participants for various cervical dilatations. Out of 1500 examinations, accurate estimates were seen in 63%. The accuracy declined for dilatations from 5 to 9cm compared to lower dilatation values from 1 to 4cm. The exception to this was full dilatation (10cm) where percentage accuracy increased again (Table 6).

**Table 6:** Percentage accuracy of the participants for various cervical dilatations.

DILATATION VALUE( IN CM)	PERCENT ACCURATE OBSERVATIONS
1	100
2	91
3	81
4	61
5	48
6	48
7	40
8	47
9	45
10	67

Feedback was taken through a questionnaire from the participants about the simulation training model. The overall feedback was positive, and they found the whole training and assessment to be a unique learning experience and thought that it improved their understanding of estimation of cervical dilatation and fetal station.

## Discussion

This study has shown the importance and usefulness of simulation models for training in assessing cervical dilatation and station to assess labour progress accurately. This is the first study comparing the accuracy of assessing both cervical dilation and fetal station between subjectively trained postgraduate trainees, senior registrars and consultants working in the department of Obstetrics and Gynaecology and undergraduate MBBS students who were trained on the vaginal simulation model. The test was performed across the full range of cervical dilatations and fetal stations on the simulation model. The improving accuracy in assessment by increasing years of experience of consultants suggests that vaginal assessment of labour progress is a skill that develops with training and experience. The only exception to this result is the lower accuracy of senior postgraduate trainees than the junior trainees. This could be the varying subjective findings of their seniors from whom they learnt vaginal assessment during their postgraduate training. Hence, it becomes essential to learn on a standard model with accurate findings.

The greater accuracy of a model trained group than a subjectively trained group for lower dilatations, i.e. 1 to 6cm, than greater dilatations, i.e. 7 to 10cm, is interesting and has a possible explanation. At lower dilatations, the examining middle and index fingers are closely opposed or slightly apart, making estimation relatively easier. Proprioceptive skill comes into play at higher dilatations, requiring estimation of the distance between the examining fingers, making it more difficult.

Also, in this study, a trained model group had better accuracy than the subjectively trained group for all fetal station values except stations 0 and +1. The possible explanation for this could be that assessment of these two stations is relatively easier due to their proximity to ischial spines. Hence, it was assessed accurately by both groups. The accurate assessment of the other station values requires proper training and practice. Therefore the trained model group performed better owing to the objective training they were given on the simulation model.

Tuffnel et al. used a carefully designed set of cervical set simulators showing dilatation and effacement, and a comparison was made between observation of midwives and obstetricians. They found accurate estimates in 49% of 360 examinations and that inaccuracies were greatest at 5 to 7cm of dilatations [3]. The findings of our study are similar in that inaccuracy was most significant at 5 to 9cm. Phelps et al. used measured sections of hard polyvinyl chloride pipe and found accurate estimates in 56% of 1574 examinations, and they observed decreasing accuracy with increasing dilatations, up to 10 cm [6]. Huhn et al. first used soft models to make examinations more realistic and found accurate estimates in only 19% of 360 examinations, followed by which they also used hard models and then reported 54% accuracy, in line with the previous studies [7]. It may be speculated that the soft models were probably less realistic than they should have been. The total accurate estimates in our study were 63% of 1500 examinations which was higher than all these studies. All these previous studies used simulation models to only assess the accuracy of the cervical assessment. No training was given on these models to the participants. Hence the drawback of these studies was that no conclusions could be drawn about the usefulness of these models for training obstetricians and students.

Buchmann & Libhaber performed an independent cervical assessment. The assessment used as a standard was performed by the experienced Obstetric consultant, the researcher, and consultants and registrars did another assessment at various levels of training. There was 49.2 % agreement between the findings of the researcher and the observers. In their study, inaccuracy of assessment was more after 5 cm dilatation, similar to the findings of our study [2]. The main drawback in their study was the absence of a gold standard value and the reliance on the researcher's estimate as being correct. The accurate standard values in our simulation model help to make observations and comparisons reliable and genuine.

Conventionally, assessing a patient's cervical dilatation is first performed by a senior in Obstetrics and Gynaecology. Then the senior informs the juniors of the cervical dilatation value just examined by him/her. Next, the junior is asked to perform a per vaginal examination of the patient. The junior then commit to memory the relationship between the examination just performed and the particular cervical dilatation value informed by the senior. Conventional training techniques like these transfer the subjective impression of one person to another without facilitating tactile guidance about the actual cervical dilatation value. Every postgraduate trainee of Obstetrics and Gynaecology undergoes such training without forming his/her judgement. Such a technique is far from perfect and is likely to result in the Student having an inaccurate judgement of the cervical dilatation assessments. Also, when a series of students examine the patients internally as a part of training, there is physical discomfort to the patient and a risk of infection to the patient due to repeated per vaginal examinations. These ethical issues will be minimized by using simulation training models like the one used in our study.

Though this simulation training model may not precisely mimic the situation of the cervix in vivo, it would surely give a palpatory learning experience to students about the cervical dilatation and levels of the fetal station during their training period. This model can be considered an in vitro training device to improve the trainees' understanding of cervical dilatation and fetal station so that the number of examinations and time required on live patients to develop the accurate skill of judgment will be minimum.

## Conclusions

This study shows that training on the simulation model will improve trainees' accuracy in their formative years and help in the betterment of their skill in the judgement of cervical dilatation and fetal station. Such a training model could be a valuable addition and should be part of the routine undergraduate and postgraduate teaching curriculum.

## References

[1] Downe S, Gyte GML, Dahlen HG, Singata M (2013). Routine vaginal examinations for assessing progress of labour to improve outcomes for women and babies at term. Cochrane Database Syst Rev.

[2] Buchmann EJ, Libhaber E (2007). Accuracy of cervical assessment in the active phase of labour. BJOG.

[3] Tufnell DJ, Bryee F, Johnson N, Lilford RJ (1989). Simulation of cervical changes in labour: reproducibility of expert assessment. The Lancet.

[4] Bedwell C, Levin K, Pett C, Lavender DT (2017). A realist review of the partograph: when and how does it work for labour monitoring?. BMC Pregnancy Childbirth.

[5] Shinde KK, Bangal VB, Singh RK (2012). Study of course of labour by using modified WHO partograph. Int J Biomed Adv Res..

[6] Phelps JY, Higby K, Smyth MH, Ward JA, Arredondo F, Mayer AR (1995). Accuracy and intraobserver variability of simulated cervical dilatation measurements. Am J Obstet Gynecol.

[7] Huhn KA, Brost BC (2004). Accuracy of simulated cervical dilation and effacement measurements among practitioners. Am J Obstet Gynecol.

